# The Role of Adiponectin in the Resolution of Male-Obesity-Associated Secondary Hypogonadism after Metabolic Surgery and Its Impact on Cardiovascular Risk

**DOI:** 10.3390/biomedicines10082000

**Published:** 2022-08-17

**Authors:** Pilar Cobeta, Roberto Pariente, Alvaro Osorio, Marta Marchan, Marta Cuadrado-Ayuso, David Pestaña, Julio Galindo, José I. Botella-Carretero

**Affiliations:** 1 Department of Anesthesiology, Hospital Universitario Ramón y Cajal, 28034 Madrid, Spain; 2 Instituto Ramón y Cajal de Investigación Sanitaria—IRyCIS, 28034 Madrid, Spain; 3 Department of Immunology, Hospital Universitario Ramón y Cajal, 28034 Madrid, Spain; 4 Department of Angiology and Vascular Surgery, Hospital Universitario Ramón y Cajal, 28034 Madrid, Spain; 5 Department of Endocrinology and Nutrition, Hospital Universitario Ramón y Cajal, 28034 Madrid, Spain; 6 Department of General and Digestive Surgery, Hospital Universitario Ramón y Cajal, 28034 Madrid, Spain

**Keywords:** male hypogonadism, testosterone, adiponectin, obesity, carotid intima-media, metabolic surgery

## Abstract

Male-obesity-associated secondary hypogonadism (MOSH) is a very prevalent entity that may resolve after marked weight loss. Adiponectin (APN) is an adipokine with anti-inflammatory properties that regulates metabolism. Low-circulating APN is associated with obesity, diabetes, and cardiovascular risk, along with circulating testosterone. We aimed to evaluate APN changes in men with MOSH (low circulating free testosterone (FT) with low or normal gonadotropins) and without it after metabolic surgery. We look for their possible association with cardiovascular risk measured by carotid intima-media thickness (cIMT). We included 60 men (20 submitted to lifestyle modification, 20 to sleeve gastrectomy, and 20 to gastric bypass) evaluated at baseline and 6 months after. The increase in APN at follow-up was reduction in patients with persistent MOSH (*n* = 10) vs. those without MOSH (*n* = 30) and MOSH resolution (*n* = 20), and the former did not achieve a decrease in cIMT. The increase in APN correlated positively with FT (r = 0.320, *p* = 0.013) and inversely with cIMT (r = −0.283, *p* = 0.028). FT inversely correlated with cIMT (r = −0.269, *p* = 0.038). In conclusion, men without MOSH or with MOSH resolution showed a high increase in APN after weight loss with beneficial effects on cIMT. Those without MOSH resolution failed to attain these effects.

## 1. Introduction

Male-obesity-associated secondary hypogonadism (MOSH) is a very prevalent entity ranging from 45–75% in men with moderate to severe obesity, as previously shown [1,2]. MOSH may resolve after the sustained and marked weight loss attained after metabolic surgery, and this occurs in parallel with the amelioration of insulin resistance and the resolution of other metabolic disorders [2,3]. Adipose tissue excess and dysfunction appear to contribute to androgen deficiency in men by effects that involve mainly the hypothalamic–pituitary level [4]. Obesity is associated with a state of both insulin and leptin resistance, which in turn reduces the release of kisspeptin at the hypothalamus, resulting in the inhibition of gonadotropin secretion [5,6]. This may be due to decreased hypothalamic Kiss1 expression, a potent regulator of gonadotropin release. As the Kiss1 neurons express leptin receptors, the Kiss1 system may provide a bridge between metabolic regulation and fertility [7]. These physio–pathological factors are reversed in the majority of patients with MOSH after metabolic surgery, increasing the circulating concentrations of total and free testosterone, inhibin B, and kisspeptin, whereas fasting insulin and leptin decrease [8,9].

Apart from inducing gonadal dysfunction, obesity has major consequences in health, as it increases the risk of cancer, metabolic diseases such as diabetes mellitus (DM), and cardiovascular diseases (CVD) [10,11]. As in the case of MOSH, metabolic surgery resolves many of those comorbidities, reducing cardiovascular risk and increasing patients’ survival [12,13,14]. Interestingly, male hypogonadism is also associated with dyslipidemia, atherosclerosis, CVD and DM, and testosterone supplementation therapy in hypogonadic men improves lipids, glycemia, and insulin sensitivity [15].

Therefore, the possibility that MOSH may have a role in the increased cardiovascular risk in men with obesity, and that its resolution after metabolic surgery contributes to its amelioration is a plausible hypothesis. Accordingly, we found previously that the significant increase in testosterone concentrations in men after metabolic surgery correlated with the decrease in carotid intima-media thickness (cIMT) [16], a strong predictor of major cardiovascular events [17,18].

Among the multiple factors that link adipose tissue excess and dysfunction with the increase in CVD, some adipokines—which are a number of inflammatory and immune mediators as well as several hormones secreted by adipose tissue—may be implicated [19]. Adiponectin (APN) is a protein hormone secreted by adipose tissue with an anti-inflammatory role that also regulates glucose and lipid metabolism [20]. Low-circulating APN concentrations are associated with obesity, type 2 DM, and CVD [21,22]. It has been shown that metabolic surgery induces an increase in APN concentrations, which drives an improvement in insulin sensitivity and type 2 DM-remission rates [23,24,25]. Conversely, other published data found no association of APN concentrations with diabetes or myocardial infarction after metabolic surgery [26]. Regarding cIMT, conflicting results have also been reported, with either a significant or a non-significant association between low-circulating APN and the severity of cIMT [27,28,29], so there remain some conflicting results.

As APN and its receptors are expressed by different cell types of the male gonad [30] and previous studies showed a relationship between APN and circulating testosterone [31,32], we aimed to evaluate their changes in men after metabolic surgery and their possible association with cIMT.

## 2. Materials and Methods

### 2.1. Patients and Study Design

We included sixty men with severe obesity and high cardiovascular risk (according to the American Heart Association) [33]. Forty of them were submitted to metabolic surgery (20 to Roux en Y gastric bypass, RYGB, and 20 to sleeve gastrectomy, SG). The other 20 patients were treated with lifestyle modifications. This was not a randomized study, and the indication for each surgical technique was in accordance with our center’s protocol. The latter is in line with current international guidelines and allocates patients with more metabolic complications to RYGB. The main characteristics of the employed surgical techniques have been reported previously [16,34].

Exclusion criteria included severe psychiatric condition or substance abuse, active neoplasia, incurable pre-existing comorbidities, testosterone treatment, and any medication which could alter circulating androgens. Patients were evaluated at baseline and 6 months after surgery or lifestyle modification.

### 2.2. Measurements

Anthropometric variables were recorded, and body mass index (BMI) was calculated. Excess body weight (EBW) was the difference between the baseline body weight and the ideal weight, the latter considered to be the weight corresponding to a BMI of 25 kg/m^2^. Excess weight loss (EWL) was calculated as the percentage of weight loss attained from baseline EBW.

APN was measured by ELISA in serum samples in duplicate with a commercial kit (Human adiponectin ELISA kit, Thermofisher Scientific, BenderMedSystems GmbH, Vienna, Austria) with an analytical sensitivity of 100 pg/mL and an intra- and inter-assay CVs of 3.5% and 5.8%, respectively.

Assays and reference ranges for total testosterone (TT), sex hormone–binding globulin (SHBG), insulin, and lipids profiles were previously reported [1]. Normal ranges were 300–900 ng/dL for TT and 225–635 pmol/L for free testosterone (FT), which was calculated using the Vermeulen formulae [1]. Reference ranges for TT were those reported from our center laboratory, and those for FT were established from a control group of healthy men as previously reported [1]. Those patients with low FT and low or normal gonadotropins were considered as having MOSH. Moreover, cIMT was measured by ultrasonography, as previously reported [16].

### 2.3. Statistics

An a priori sample size analysis was performed with the online tool GRANMO 7.12 (https://www.imim.es/ofertadeserveis/software-public/granmo/index.html accessed on 1 February 2018). A total sample size of 24 subjects was enough to detect a mean difference of 3 µg/mL in circulating APN with a SD of 5 in the follow-up period with 1 − β = 0.80 and α = 0.05.

The results are expressed as means ± SD unless otherwise stated. The Kolmogorov–Smirnov statistic was applied to continuous variables. Logarithmic or square root transformations were used as needed to ensure normal distribution. Student *t* test or one-way analysis of variance with Tukey tests compared the central tendencies of the different groups. Mann–Whitney U test or Kruskal–Wallis test followed by Wilcoxon tests were used in the case of non-normal variables. For discontinuous variables, the χ^2^ test and Fisher’s exact test were employed.

Comparisons of continuous variables before and after bariatric surgery were performed by repeated-measure general linear model analysis, and the group of subjects was introduced as the between-subject effect. Bivariate correlation analyzed the association between two continuous variables by Pearson or Spearman’s tests.

Multivariate analysis was used to find the effects of several independent variables and their interactions on the changes in APN, FT, and cIMT and to correct for the effects of different degrees of weight loss. For this purpose, data were submitted to a full-factorial multivariate general linear model (GLM) with a type III sum of squares, comparing the main effects of the fixed factors using Roy’s largest root. We used SPSS 18 (SPSS Inc, Chicago, IL, USA), and *p* < 0.05 was considered statistically significant.

## 3. Results

### 3.1. Baseline Characteristics in Patients with and without MOSH

Sixty patients with an age of 48 ± 9y (lifestyle modification *n* = 20, age 48 ± 8y, SG *n* = 20, age 46 ± 9y, RYGB *n* = 20, age 51 ± 9y, *p* = 0.190) were evaluated. Of these, 30 patients (50%) presented MOSH at baseline, and the other 30 (50%) had normal FT. Their baseline clinical and biochemical characteristics are shown in Table 1. As expected, patients with MOSH had lower TT and FT. SHBG concentrations were also lower in patients with MOSH, and they also had higher BMI and EBW, but the baseline circulating APN did not differ (Table 1).

### 3.2. Changes at Follow-Up Depending on the Presence of MOSH and Its Resolution

When considering the resolution of MOSH after metabolic surgery, patients were categorized into three groups: those without MOSH at baseline (*n* = 30), those with MOSH at baseline who attained complete normalization of FT after the intervention (MOSH resolution, *n* = 20: after lifestyle modification *n* = 1, after SG *n* = 9, after RYGB *n* = 10), and finally those with MOSH at baseline with persistence of low FT after six months of therapy (MOSH persistence, *n* = 10: after lifestyle modification *n* = 7, after SG *n* = 3, after RYGB *n* = 0).

**Figure 1:**
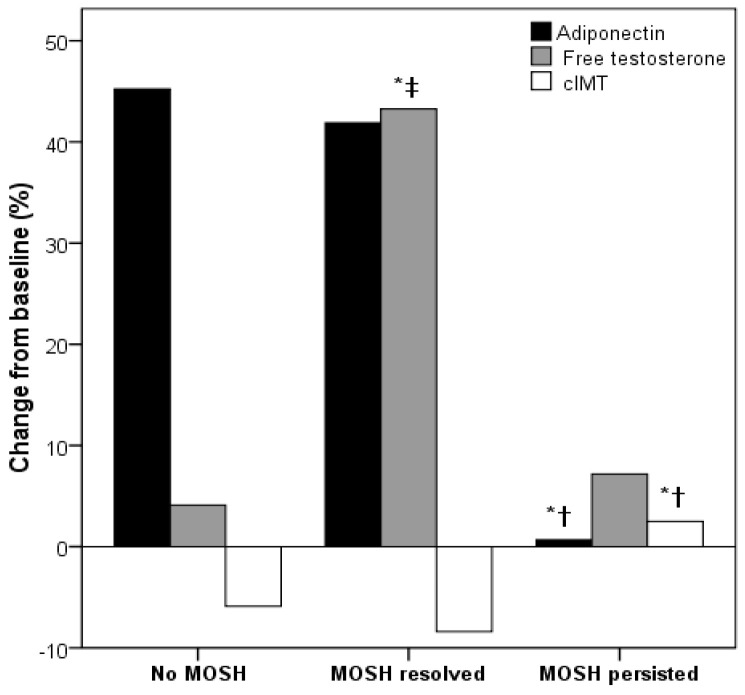
Changes in circulating adiponectin, free testosterone (FT), and carotid intima-media thickness (cIMT) in the included men depending on the presence of MOSH and its resolution. * *p* < 0.05 vs. no MOSH, † *p* < 0.05 vs. MOSH resolved, ‡ *p* < 0.05 vs. MOSH persisted.

The increase in APN at follow-up was lower in those patients with persistent MOSH compared to those without MOSH and with MOSH resolution, and the former did not achieve a decrease in cIMT at follow-up. As expected, the increase in FT was higher in those patients with MOSH resolution (Figure 1).

Regarding other changes after the intervention (Table 2), patients with persistence of MOSH showed lower EWL than the other two groups (F = 3.683, *p* = 0.031), whereas diastolic BP was lower after MOSH resolution (F = 4.680, *p* = 0.013). Conversely, changes between the three groups in systolic BP, LDL, HDL, glycemia, insulin, and HOMA-IR were not significant (F = 0.866, *p* = 0.426; F = 0.447, *p* = 0.642; F = 0.506, *p* = 0.606; F = 1.135, *p* = 0.329; F = 0.259, *p* = 0.772; and F = 2.330, *p* = 0.106, respectively) (Table 2).

### 3.3. Correlation of Adiponectin Changes at Follow-Up with Other Variables

Considering all participants, a positive correlation was found between the increase in APN and the changes in TT (r = 0.518, *p* < 0.001), SHBG (r = 0.446, *p* < 0.001), and FT (r = 0.320, *p* = 0.013), as well as a negative correlation with cIMT and BMI (Table 3). Both TT and FT also showed negative correlations with the changes in cIMT, BMI, systolic and diastolic BP, glucose, insulin, and HOMA-IR, as well as a positive correlation with the changes in HDL (Table 3).

### 3.4. Effects of the Type of Therapy

Regarding the type of therapy for obesity, the distribution of patients and their characteristics are shown in Table 4. At baseline, patients submitted to RYGB showed lower concentrations of LDL cholesterol because of a more frequent use of statins. At follow-up, the effect of metabolic surgery was higher than those of lifestyle modifications on BMI, EBW, cIMT, blood pressure, fasting glucose, and insulin (Table 4).

**Figure 2:**
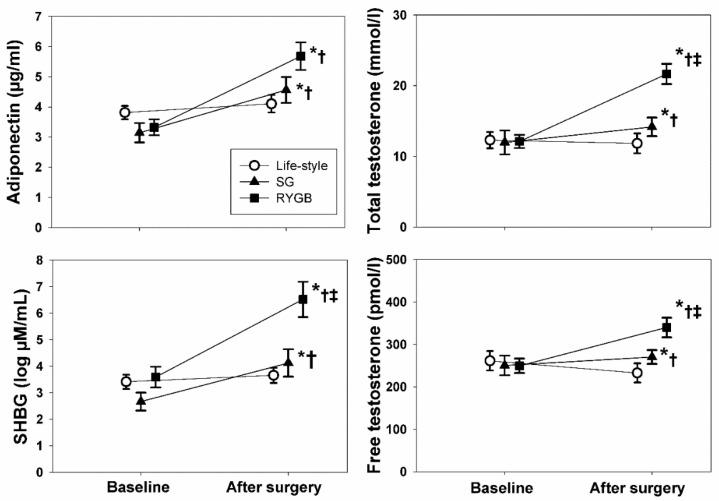
Changes in circulating adiponectin and androgens in the included men after obesity surgery. Symbols represent means and error bars represent SEMs. * *p* < 0.05 from baseline, † *p* < 0.05 vs. lifestyle modification, ‡ *p* < 0.05 vs. SG.

APN concentrations increased after metabolic surgery, more after RYGB than SG, with no changes in lifestyle modification (Wilks’ λ = 0.835, *p* < 0.001 for the interaction, *p* = 0.005 for RYGB vs. lifestyle, *p* = 0.001 for SG vs. lifestyle, *p* = 0.069 for RYGB vs. SG) (Figure 2). When correcting for the presence of diabetes, the results were similar (Wilks’ λ = 0.999, *p* = 0.803 for the interaction). TT, SHBG, and FT increased after metabolic surgery compared with lifestyle modification, and the effect of RYGB was higher (Figure 2).

### 3.5. Ancillary Analyses

Multivariate analysis was performed in order to correct for the different amount of weight loss in patients according to the presence of MOSH and its resolution and also for the different treatments (lifestyle modification and metabolic surgery). We employed a full-factorial multivariate general linear model (GLM) introducing the changes in APN, FT, and cIMT as dependent variables; the presence of MOSH and its resolution (no MOSH, MOSH resolved, MOSH persisted) and type of treatment (lifestyle modification, SG, RYGB) as fixed effects; and EWL as a covariate.

Multivariate tests showed an overall significance of the effects of MOSH and its resolution (F = 3.913, *p* = 0.014), the type of treatment (F = 3.723, *p* = 0.017), and their interaction (F = 3.683, *p* = 0.018) on the dependent variables, but not for EWL as a covariate (F = 0.691, *p* = 0.562). Estimated marginal means and pairwise comparison showed that changes in cIMT stayed significant for the comparisons between persistence of MOSH vs. no MOSH (*p* = 0.023) and vs. MOSH resolution (*p* = 0.002). Changes in APN stayed significant for the comparison between MOSH persistence vs. no MOSH (*p* = 0.025). Changes in FT stayed significant for the comparison between MOSH resolution vs. no MOSH (*p* = 0.045).

## 4. Discussion

We found that, in men with obesity after metabolic surgery, there is an increase in circulating APN concentrations, which is associated with an increase in testosterone and a decrease in cIMT. Furthermore, those men without MOSH or with MOSH resolution after weight loss showed a higher increase in APN with a reduction in cIMT, but this did not occur in those men with persistent MOSH. Our data also showed, after multivariate analysis, that both metabolic surgery and the presence of MOSH and its resolution had an interaction on these changes beyond that the effect of weight loss. To our knowledge, this is the first time that the relationship of circulating APN with the changes in cIMT in patients with or without MOSH are explored concomitantly in men with obesity.

The cIMT is a strong predictor of major cardiovascular events [17,18], and low APN concentrations seem to independently predict the progression of carotid atherosclerosis and cardiac remodeling [35,36]. However, previous data have shown conflicting results regarding the relationship between APN and cIMT; on the one hand, a 5-year prospective study with first-degree relatives of patients with type 2 DM and normal individuals could not find a significant association [16]. Further, the prospective controlled Swedish Obese Subjects study [26], with more than 3299 patients, showed that the observed 2-year increases in APN after metabolic surgery were not associated with the risk of myocardial infarction or stroke [26]. In agreement, we were not able to demonstrate an association between circulating APN and cIMT in a prospective study with women evaluated at baseline and 1 year after obesity surgery, albeit the observed increase in APN was associated with a reduction in insulin resistance [18].

On the other hand, there are consistent published data showing that the increase in circulating APN after metabolic surgery induces beneficial metabolic changes, such as the reduction in insulin resistance, the resolution of type 2 DM [23,25,37,38], improvements in lipid profiles, and the reduction of inflammatory markers [39,40,41,42]. In agreement, a recent meta-analysis found an inverse association between APN levels and cIMT [29], and, in the Northern Manhattan Study that included a cohort of 1522 individuals, low APN was associated with increased cIMT, supporting the protective role for APN in atherosclerosis [27]. Therefore, according to these data and the results of our present study, it is plausible that the relationship of the changes in APN after weight loss with cIMT may exhibit sexual dimorphism, and, in men with obesity, its actions through the changes in circulating testosterone might have a significant effect on cIMT.

Regarding the relationship of APN with the male gonadal axis, there is evidence of the expression of APN receptors by Leydig cells, spermatozoa, and epididymis [30]. Functionally, APN can regulate the expression of different steroidogenic genes [43], and, in addition, it has been shown to promote spermatogenesis and sperm maturation [30]. However, previous studies exploring the association of circulating APN with androgens in men have yielded conflicting results. Elsaied et al. described a positive correlation in 87 patients, 58 with type 2 DM, and 29 nondiabetics [44], and Rasul et al. found the same association in 62 elderly diabetic men [45]. Conversely, Frederiksen et al. reported a decrease in APN concentrations after 6 months of testosterone therapy in a group of 38 aged men [46], and similar findings were shown in another two studies [32,47]. Therefore, the associations of APN and androgens are complex and may be confounded by the degree of adiposity.

Excessive amounts of adipose tissue, particularly in the visceral depot, favors the development of hypogonadism through several mechanisms. One is the increased aromatase activity that converts testosterone to estrogens that may lead to further peripheral fat accumulation, both by increasing the concentration of estrogens and reducing LH-induced testosterone production [48]. Another mechanism is the increased release of pro-inflammatory cytokines by dysfunctional adipose tissue that suppresses the release of gonadotropin hormones, particularly LH, with a concomitant decrease in testosterone production [49]. This central effect is mainly due to the reduced responsiveness of hypothalamic neurons to kisspeptins, as mentioned earlier, but the decrease of testosterone may also be due to the direct effect of the pro-inflammatory mediators on Leydig cells [50]. The third mechanism is the alterations in adypokine release, mainly hyperleptinemia with leptin resistance, which has been shown to induce apoptosis in Leydig cells and reduce sperm count and motility [51]; and also, as stated above, a reduction in APN that may directly control the function of Leydig cells through the activation of key protein and enzymes involved in androgen synthesis [52]. All these physio–pathological mechanisms may lead to the occurrence of MOSH, with its prevalence being dependent on the degree of adiposity, from 10% when BMI is between 30–34.9 to almost an 80% when BMI is above 50 [53].

Specifically, the association of MOSH with cardiovascular risk has not been completely established yet; however, clinical and epidemiological studies found that male hypogonadism was associated with dyslipidemia, atherosclerosis, CVD, and DM [54,55]. Further, testosterone supplementation therapy in hypogonadic men showed improvements in lipids, glycemia, and insulin sensitivity [15]. Testosterone exerts vasorelaxation at the vascular level through a rapid non-genomic action [55], and this may be a key factor beyond changes in metabolism to achieve a beneficial cardiovascular effect. Recently, a systematic review and meta-analysis evaluated the effect of testosterone replacement therapy in men with obesity who had low testosterone levels [56]. It showed that testosterone therapy slightly improved the lean body mass and LDL but with no effect on blood pressure, and the effects on cardiovascular events, HbA1c, and quality of life were unclear.

Metabolic surgery is a very effective treatment for MOSH [2], and it can be hypothesized that its resolution might reduce cardiovascular risk far beyond that of weight loss. In agreement, we found a significant increase in FT concentrations in men after metabolic surgery which inversely correlated with blood pressure, lipids, and insulin resistance and positively with APN. However, more importantly, a novel result is that men with persistent MOSH had a low increase in APN with no reduction in cIMT after weight loss, whereas those men without MOSH or with MOSH resolution after weight loss showed a higher increase in APN with a reduction in cIMT. These effects were demonstrated after correction for different amounts of weight loss in the multivariate analysis that showed an interaction of both MOSH resolution and the type of therapy for weight loss.

Our present study has the major limitation of the lack of randomization in the allocation of the patients to the different groups of interventions. Another important limitation is that causality cannot be ascertained. So, whether weight loss is the causal effect on MOSH resolution and improvement in cIMT or, conversely, whether the persistence of MOSH limits weight loss and has a beneficial effect on cIMT cannot be addressed by our design. In addition, the generalizability of the results is limited, because we evaluated the patients after a short-term period of 6 months, so the long-term beneficial effects of the changes in APN and testosterone on cIMT and cardiovascular events could not be evaluated.

## 5. Conclusions

Men without MOSH or with MOSH resolution showed a high increase in APN after weight loss and exhibited beneficial effects on cIMT. Those without MOSH resolution failed to attain the latter. Metabolic surgery induced a significant increase in circulating APN, which paralleled an increase in FT, and these changes were associated with beneficial effects in blood pressure, lipid profiles, and insulin sensitivity. Future studies are needed to confirm our results.

## Figures and Tables

**Table 1:** Characteristics of men with and without MOSH at baseline.

	MOSH (*n* = 30)	No MOSH (*n* = 30)
Age (y)	47 ± 9	49 ± 8
BMI (Kg/m^2^)	45.8 ± 6.5	42.4 ±5.4 *
EBW (kg)	64.6 ± 20.3	54.3 ± 18.0 *
cIMT (mm)	0.64 ± 0.11	0.68 ± 0.12
Systolic BP (mmHg)	136 ± 14	137 ± 19
Diastolic BP (mmHg)	82 ± 11	83 ± 11
LDL (mmol/L)	3.1 ± 0.8	3.0 ± 0.9
HDL (mmol/L)	1.0 ± 0.8	1.0 ± 0.4
Glucose (mmol/L)	6.6 ± 2.3	6.2 ± 1.8
Insulin (mU/L)	24 ± 14	23 ± 14
HOMA-IR	6.8 ± 4.7	7.3 ± 7.4
TT (ng/dL)	253 ± 163	440 ± 105 *
SHBG (µmol/dL)	24.5 ± 12.5	32.6 ± 13.6 *
FT (pmol/L)	179 ± 46	324 ± 68 *
APN (µg/mL)	3.7 ± 1.7	3.9 ± 1.5

MOSH: male-obesity-associated secondary hypogonadism, BMI: body mass index, EBW: excess body weight, cIMT: carotid intima-media thickness, BP: blood pressure, LDL: low density lipoprotein cholesterol, HDL: high density lipoprotein cholesterol, HOMA-IR: insulin resistance calculated by the homeostatic model assessment, TT: total testosterone, SHBG: sex hormone binding globulin, FT: free testosterone, APN: adiponectin. * *p* < 0.05 between groups.

**Table 2:** Changes at follow-up depending on the presence of MOSH and its resolution.

	No MOSH (*n* = 30)	MOSH Resolved (*n* = 20)	MOSH Persisted (*n* = 10)
EWL (kg)	44.5 ± 42.0	50.6 ± 30.7	13.4 ± 27.5 *^,†^
BMI (Kg/m^2^)	−8.1 ± 8.2	−9.8 ± 6.6	−1.9 ± 6.4 *^,†^
Systolic BP (mmHg)	−5.4 ± 25.2	−11.2 ± 9.6	−1.6 ± 18.4
Diastolic BP (mmHg)	−1.3 ± 14.1	−10.9 ± 11.4 *^,^^‡^	−0.7 ± 4.7
LDL (mmol/L)	−0.08 ± 0.88	−0.14 ± 1.01	−0.12 ± 0.49
HDL (mmol/L)	−1.05 ± 0.37	−0.97 ± 0.25	−0.97 ± 0.28
Glucose (mmol/L)	−0.6 ± 2.1	−1.0 ± 2.3	0.2 ± 0.5
Insulin (mU/L)	−7.5 ± 11.3	−10.1 ± 10.3	−8.5 ± 17.6
HOMA-IR	−1.1 ± 7.1	−3.2 ± 3.8	4.9 ± 14.8

MOSH: male-obesity-associated secondary hypogonadism, EWL: excess weight loss, BMI: body mass index, BP: blood pressure, LDL: low density lipoprotein cholesterol, HDL: high density lipoprotein cholesterol, HOMA-IR: insulin resistance calculated by the homeostatic model assessment. * *p* < 0.05 vs. no MOSH, ^†^ *p* < 0.05 vs. MOSH resolved, ^‡^ *p* < 0.05 vs. MOSH persisted.

**Table 3:** Correlation coefficient of changes in adiponectin and androgens with other variables.

	Δ APN	Δ TT	Δ SHBG	Δ FT
Δ cIMT	−0.283 *	−0.428 *	−0.347 **	−0.269 *
Δ BMI	−0.266 *	−0.583 **	−0.691 **	−0.337 **
Δ Systolic BP	−0.211	−0.394 **	−0.205	−0.419 **
Δ Diastolic BP	−0.059	−0.291 *	−0.185	−0.342 **
Δ LDL	−0.230	−0.099	−0.060	−0.035
Δ HDL	0.046	0.442 **	0.355 **	0.324 *
Δ Glucose	−0.135	−0.421 **	−0.273 *	−0.379 **
Δ Insulin	−0.158	−0.516 **	−0.405 **	−0.399 **
Δ HOMA-IR	−0.157	−0.508 **	−0.378 **	−0.395 **

Δ: changes of variables calculated as % of variation from baseline, APN: adiponectin, TT: total testosterone, SHBG: sex hormone binding globulin, FT: free testosterone, cIMT: carotid intima-media thickness, BMI: body mass index, BP: blood pressure, LDL: low density lipoprotein cholesterol, HDL: high density lipoprotein cholesterol, HOMA-IR: insulin resistance calculated by the homeostatic model assessment. * *p* < 0.05, ** *p* < 0.01.

**Table 4:** Baseline and follow-up characteristics regarding the type of therapy for obesity.

	Lifestyle (*n* = 20)		SG (*n* = 20)		RYGB (*n* = 20)	
	Baseline	6 Months	Baseline	6 Months	Baseline	6 Months
BMI (Kg/m^2^)	44.0 ± 5.4	45.2 ±7.1	45.0 ±6.9	33.2 ± 4.1 *^,†^	43.7 ± 7.2	31.6 ± 6.5 *^,†^
EBW (kg)	59.4 ± 17.4	3.7 ± 14.1	61.6 ± 21.2	57.4 ± 18.2 *^,†^	56.7 ± 20.8	69.4 ± 26.2 *^,†^
cIMT (mm)	0.66 ± 0.10	0.67 ± 0.11	0.65 ± 0.11	0.60 ± 0.09 *^,†^	0.66 ± 0.13	0.60 ± 0.12 *^,†^
Systolic BP (mmHg)	145 ± 16	151 ± 12	141 ± 17	130 ± 12 *^,†^	144 ± 16	129 ± 17 *^,†^
Diastolic BP (mmHg)	87 ± 9	89 ± 8	85 ± 11	84 ± 8^†^	87 ± 11	76 ± 12 *^,†^
LDL (mmol/L)	3.0 ± 0.8	3.2 ± 0.6	3.0 ± 0.9	2.9 ± 0.8	2.1 ± 0.8 ^†,‡^	1.9 ± 0.7
HDL (mmol/L)	1.1 ± 0.4	1.0 ± 0.3	1.0 ± 0.2	1.2 ± 0.2 *^,†^	1.0 ± 0.2	1.2 ± 0.4 *^,†^
Glucose (mmol/L)	6.6 ± 2.3	7.0 ± 2.9	5.9 ± 1.9	5.4 ± 0.9 ^†^	7.2 ± 3.3	5.5 ± 1.7 *^,†^
Insulin (mU/L)	30 ± 16	27 ± 14	19 ± 9†	10 ± 6 *^,†^	21 ± 14 ^†^	7 ± 3 *^,†^
HOMA-IR	9.5 ± 8.0	15.2 ± 28.6	5.1 ± 3.1†	2.4 ± 1.6	6.4 ± 5.7 ^†^	3.2 ± 5.8

SG: sleeve gastrectomy, RYGB: Roux-en-Y gastric bypass, BMI: body mass index, EBW: excess body weight, DM: diabetes mellitus, cIMT: carotid intima-media thickness, BP: blood pressure, LDL: low density lipoprotein cholesterol, HDL: high density lipoprotein cholesterol, HOMA-IR: insulin resistance calculated by the homeostatic model assessment, TT: total testosterone, SHBG: sex hormone binding globulin, FT: free testosterone. * *p* < 0.05 from baseline, ^†^ *p* < 0.05 vs. controls, ^‡^ *p* < 0.05 vs. SG.

## Data Availability

Restrictions apply to the availability of data generated or analyzed during this study to preserve patient confidentiality or because they were used under license. The corresponding author will, on request, detail the restrictions and any conditions under which access to some data may be provided.
